# Genetic and Epigenetic Components in the Pathogenesis of Adenomyosis and Endometriosis in Adolescents

**DOI:** 10.3390/biomedicines13122988

**Published:** 2025-12-05

**Authors:** Mario Palumbo, Luigi Della Corte, Mario Ascione, Giuseppe D’Angelo, Dario Colacurci, Giorgio Maria Baldini, Massimiliano Pellicano, Pierluigi Giampaolino, Giuseppe Bifulco

**Affiliations:** 1 Department of Public Health, School of Medicine, University of Naples “Federico II”, 80131 Naples, Italy; mario.ascione@unina.it (M.A.); giuseppe.dangelo3@unina.it (G.D.); dario.colacurci@unina.it (D.C.); pellican@unina.it (M.P.); pierluigi.giampaolino@unina.it (P.G.); giuseppe.bifulco@unina.it (G.B.); 2 Department of Neuroscience, Reproductive Sciences and Dentistry, School of Medicine, University of Naples “Federico II”, 80131 Naples, Italy; luigi.dellacorte@unina.it; 3 Department of Biomedical Sciences and Human Oncology, University of Bari “Aldo Moro”, Piazza Giulio Cesare 11, 70124 Bari, Italy; giorgio.baldini@uniba.it

**Keywords:** endometriosis, adenomyosis, adolescents, genetics, epigenetics, heritability, estrogen receptor, WNT4, GWAS, gene–environment interaction

## Abstract

**Background:** Adenomyosis and endometriosis are complex, estrogen-dependent gynecological conditions increasingly diagnosed in adolescents. While traditionally considered diseases of reproductive-age women, emerging evidence suggests a possible developmental origin in some cases, with genetic and epigenetic susceptibility playing a central role. Understanding the contribution of hereditary and molecular factors in adolescent-onset forms may offer insights into early pathogenesis, personalized risk stratification and tailored prevention strategies. **Objectives:** The objectives of this study were to explore the current evidence supporting a genetic contribution to the development of adenomyosis and endometriosis in adolescents and to identify specific genetic variants, molecular pathways and epigenetic mechanisms potentially involved in early-onset disease. **Methods:** A narrative literature review was conducted using PubMed and Scopus databases up to September 2025. Studies investigating the genetic basis of adenomyosis and endometriosis in adolescents, including familial aggregation, twin studies, GWAS and candidate gene analyses, were included. **Results:** Evidence from familial clustering and twin studies suggests a significant heritable component in both conditions. Genome-wide association studies have identified susceptibility loci, particularly involving *WNT4*, *VEZT* and *ESR1*, that may be relevant to adolescent-onset disease. Candidate gene studies further highlight the roles of estrogen signalling, inflammatory pathways, extracellular matrix remodelling and emerging epigenetic alterations, including aberrant DNA methylation and chromatin remodelling, which may influence early lesion development. However, most data are derived from adult cohorts, with limited adolescent-specific analyses. **Conclusions:** Genetic and epigenetic predispositions appear to contribute significantly to the pathogenesis of endometriosis and possibly adenomyosis in adolescents. Further studies targeting early-onset disease are needed to unravel developmental mechanisms and gene–environment interactions unique to this population.

## 1. Introduction

### 1.1. Adenomyosis and Endometriosis in Adolescents

Adolescent-onset endometriosis and adenomyosis are increasingly recognized as genuine clinical entities rather than rare exceptions, yet they remain substantially underdiagnosed [1,2]. A growing body of evidence suggests that genetic susceptibility may lower the threshold for disease manifestation during adolescence, particularly during the profound endocrine and immunological transitions of puberty [3,4]. Variants in key loci such as *WNT4* [5], *ESR1* [6], *VEZT* [7], *FSHB* [8], and genes regulating estrogen metabolism, inflammatory reactivity and Müllerian development may predispose genetically vulnerable girls to earlier lesion formation once exposed to pubertal hormonal surges or environmental triggers; in parallel, emerging epigenetic mechanisms such as DNA methylation changes in steroidogenic, inflammatory and Müllerian-development pathways may further modulate gene expression during puberty, amplifying susceptibility to early lesion initiation [9,10,11].

Whether individuals carrying specific susceptibility variants exhibit an earlier onset of endometriosis (and possibly adenomyosis), compared with those carrying wild-type or alternative alleles, remains largely unknown [5,6]. Although genetic susceptibility clearly contributes to disease vulnerability, genotype–phenotype correlations with age at symptom onset have not yet been systematically investigated [7,8]. Future studies should therefore stratify patients by age of onset to determine whether particular variants are associated with adolescent-onset phenotypes [9,10].

This developmental framework provides a compelling biological basis for why symptoms can emerge soon after menarche in a subset of adolescents carrying high-risk genotypes [11,12].

In parallel, diagnostic capabilities have markedly improved in recent years, largely due to the refinement of endocavitary ultrasound techniques, including transvaginal and transrectal approaches, which offer high-resolution imaging even in very young patients in whom transvaginal scanning may not be feasible [13,14].

The implementation of standardized imaging protocols, such as the International Deep Endometriosis Analysis (IDEA) consensus for endometriosis mapping and the MUSA (Morphological Uterus Sonographic Assessment) criteria for adenomyosis, has enhanced the accuracy, reproducibility and early detection of uterine and extra-uterine disease, even in younger patients [15,16]. These structured sonographic frameworks support a more systematic evaluation of pelvic anatomy, enabling trained second-level sonographers to identify subtle, early-stage lesions that were previously overlooked [17].

Despite these advances, dysmenorrhea, heavy menstrual bleeding and chronic pelvic pain in adolescents are frequently normalized, contributing to prolonged diagnostic delays and inappropriate reassurance [18,19]. Contemporary literature consistently reports an average diagnostic delay of 7–10 years for endometriosis across age groups, with several systematic reviews demonstrating ranges between 5 and 12 years [19,20]. This delay, compounded by the misconception that these conditions do not occur in adolescents and by the absence of dedicated diagnostic pathways, facilitates disease progression and unnecessary suffering [21,22].

For these reasons, the present review also serves as a call to action and an alert against misdiagnosis. Clinicians should regard severe or refractory dysmenorrhea, HMB or chronic pelvic pain in adolescents as red flags warranting timely evaluation by experienced providers and second-level sonographers with specific expertise in endometriosis and adenomyosis (i.e., clinicians with dedicated 5-year training in the diagnosis of endometriosis and adenomyosis and performing an average of at least 500 ultrasound examinations per year) [23]. Early recognition is particularly critical in genetically predisposed patients in whom gene–hormone–environment interactions may accelerate the trajectory of disease [24,25]. By integrating genetic, epigenetic and developmental evidence, including chromatin remodelling, aberrant methylation and transcriptional dysregulation, the present review underscores how inherited and acquired molecular alterations may jointly shape early disease trajectories in the peri-menarchal and adolescent period [10,11]. This manuscript aims to raise awareness among gynecologists, pediatric–adolescent specialists and imaging experts, reinforcing the need for informed diagnostic strategies that prevent years of avoidable delay and improve long-term reproductive and quality-of-life outcomes [26]. Finally, obtaining a thorough family history regarding menstrual bleeding patterns in the patient’s mother, aunts and sisters can help better identify potential familial correlations. Overall, this work provides an overview of the potential association between the genetic and epigenetic background of affected women and the development of endometriosis and adenomyosis.

### 1.2. Genetic Basis of Gynecological Diseases

The understanding that complex gynecological diseases may have a substantial genetic component has catalyzed extensive research aimed at identifying heritable risk factors and the molecular pathways that underlie these disorders [27,28]. Endometriosis, in particular, has been one of the most extensively investigated conditions in this context [29]. Evidence from familial aggregation studies and twin cohorts consistently indicates that up to 50% of disease susceptibility may be genetically determined, highlighting a remarkably strong heritable component [30]. Although adenomyosis has received comparatively less attention, emerging data similarly suggest familial clustering and potential genetic overlap with endometriosis, supporting the hypothesis that the two disorders may share common biological roots [31,32].

The advent of genome-wide association studies (GWAS) has substantially expanded our understanding of the genetic architecture of endometriosis [30]. Several robust susceptibility loci have been identified, including *WNT4* [5], *ESR1* [6], *FSHB* [8] and *VEZT* [7], which are implicated in hormonal regulation, inflammatory signalling, cell adhesion and reproductive tract development [33,34]. Many of these pathways are biologically active during puberty, making them particularly relevant for explaining why a subset of patients may develop symptoms early in adolescence [35,36]. Complementing GWAS, candidate gene studies have uncovered clinically meaningful polymorphisms affecting estrogen receptor activity, steroid biosynthesis, local estrogen metabolism, immune responsiveness and extracellular matrix remodelling, processes central to the initiation and persistence of ectopic endometrial tissue [24,30].

Despite these advances, the genetic determinants of early-onset disease remain poorly defined [36]. Adolescents represent a unique biological population in whom genetic background interacts with pubertal hormonal maturation, immune system reprogramming and environmental exposures during critical developmental windows [37]. These interactions may generate distinct molecular signatures that differ from those observed in adult-onset disease. A deeper understanding of the genetic and epigenetic drivers of adolescent-onset adenomyosis and endometriosis may therefore provide novel insights into early pathogenic mechanisms and ultimately support improved risk stratification, timely diagnosis and personalized prevention or treatment strategies [37,38].

## 2. Materials and Methods

A comprehensive literature search was performed using the following electronic databases: MEDLINE, EMBASE, Web of Science, PubMed, and the Cochrane Library.

The search strategy employed a combination of Medical Subject Headings (MeSH) and free-text terms, including: “endometriosis,” “adenomyosis,” “adolescents,” “genetics,” “genetic susceptibility,” “genome-wide association study,” “familial aggregation,” and “gene polymorphism.” Boolean operators (“AND,” “OR”) were used to optimize the sensitivity and specificity of the search. The time frame considered was from January 2010 to September 2025.

Two reviewers (MP and LDC) independently screened all titles and abstracts retrieved through the initial search. All types of study designs were considered eligible, including observational studies, case–control studies, cohort studies, twin studies, GWAS, and genetic association studies. Articles deemed potentially relevant were selected for full-text review. Inclusion criteria required that studies be published in English and report original data related to the genetic or molecular mechanisms underlying adenomyosis and/or endometriosis, with particular focus on adolescent populations.

Disagreements during the selection process were resolved through discussion, and when necessary, a third reviewer (MA) was consulted to achieve consensus. In addition, two reviewers (GD and DC) manually reviewed the reference lists of all included articles to identify any studies that may have been missed in the database search.

Articles were excluded if they met any of the following criteria: (1) focused exclusively on in vitro or animal model studies without translational relevance to human disease; (2) were conference proceedings, abstracts, or non-peer-reviewed publications; or (3) did not report specific findings related to genetic or molecular aspects of adenomyosis or endometriosis.

Relevant data from each included study were extracted and synthesized narratively, with particular attention to (i) the genes or genetic loci investigated; (ii) their molecular functions and biological pathways; (iii) the pathophysiological context of adolescent disease; and (iv) the clinical relevance of the findings. Priority was given to original studies providing functional or mechanistic insights into the genetic and epigenetic underpinnings of early-onset adenomyosis and endometriosis. In contrast, studies limited to purely descriptive associations, such as SNP association studies without functional validation, were excluded to enhance the translational relevance of the review.

## 3. Genetic Contributions

### 3.1. Familial Aggregation and Heritability

Familial clustering of endometriosis has long been recognized as a strong indicator of genetic susceptibility. Multiple studies have demonstrated a markedly increased risk of disease among first-degree relatives of affected women, with odds ratios reported between 4.3 and 7.6 [24,30]. This pattern has been observed across diverse populations and ethnic groups, supporting a genuine heritable contribution rather than shared environmental exposure alone [39]. Importantly, early-onset presentations and more severe phenotypes, including deep infiltrating endometriosis (DIE) and multifocal or extensive disease, have been reported more frequently in familial cases, suggesting that a higher genetic load may influence both the severity and timing of symptom onset [39,40].

Moreover, it should be emphasized that several familial, menstrual, and reproductive characteristics have long been recognized as important contributors to endometriosis risk, shaping both susceptibility and the clinical timing of disease onset [41]. Among these, a positive first-degree family history remains one of the most robust predictors, highlighting the substantial heritable component of the disorder and the likelihood that predisposing genetic backgrounds cluster within families [42,43]. This familial aggregation supports a model in which inherited biological traits create a baseline vulnerability that may manifest clinically under specific hormonal or environmental conditions [44,45].

Menstrual factors also play a central role in modulating disease risk [44,45]. Early menarche, short or highly frequent menstrual cycles, and prolonged bleeding duration have all been consistently associated with an increased lifetime risk of endometriosis [46]. These features are thought to increase cumulative exposure to retrograde menstruation, promote peritoneal inflammation, and amplify estrogen-dependent tissue activity [47]. In adolescents, in whom hormonal rhythms are still maturing, such menstrual patterns may further accentuate susceptibility by intensifying the inflammatory and endocrine milieu that favours lesion establishment and persistence [47,48].

Reproductive characteristics likewise influence disease risk across the life course. Nulliparity, low parity, and delayed childbearing have repeatedly been reported as risk-enhancing factors, potentially reflecting the protective effects of pregnancy-associated anovulation and progesterone-dominant states [46]. Some epidemiological studies have also suggested that early age at first birth may contribute to risk in selected populations, although findings remain heterogeneous [47]. Together, these reproductive patterns interact with menstrual history to shape long-term exposure to ovulatory cycles, estrogen levels, and pelvic inflammatory activity, all mechanisms relevant to endometriosis pathogenesis [48,49].

In addition to these reproductive and menstrual traits, anthropometric and constitutional factors have emerged as relevant modifiers of risk [50]. Low body mass index, a reduced waist-to-hip ratio, and heightened pelvic nociceptive sensitivity have been associated with increased disease likelihood [51]. These observations underscore the complexity of endometriosis as a disorder influenced by systemic metabolic, endocrine, and neurobiological dimensions rather than reproductive physiology alone [48,49].

Collectively, these familial, menstrual, reproductive, and anthropometric factors delineate a multifactorial risk profile, in which genetic predisposition intersects with individual biological trajectories to determine not only the probability of developing endometriosis, but also the age at which the disease becomes clinically apparent [50]. This broader perspective reinforces the importance of early recognition in adolescents who present with high-risk features, particularly when symptoms are severe, refractory, or emerge shortly after menarche [51].

In contrast, familial data for adenomyosis are comparatively scarce. Nonetheless, emerging evidence from registry-based analyses and retrospective cohorts has begun to suggest familial aggregation, particularly among women diagnosed at a younger age [52]. The frequent co-occurrence of adenomyosis and endometriosis within the same family units further reinforces the possibility of shared heritable mechanisms, although differences in diagnostic timing and historical under-recognition of adenomyosis often obscure this relationship [53]. The absence of systematic, family-based studies specifically targeting adolescent populations represents a significant gap in the current literature and underscores the need for dedicated research in this area [54].

### 3.2. Biological Transitions of Adolescence and Their Implications for Endometriosis Risk

Adolescence represents a critical developmental window during which profound neuroendocrine, immunological, epigenetic, and genetic processes converge to shape reproductive maturation [45]. The activation of the hypothalamic–pituitary–gonadal (HPG) axis begins with pulsatile gonadotropin-releasing hormone (GnRH) secretion from the hypothalamus, which stimulates the anterior pituitary to release follicle-stimulating hormone (FSH) and luteinizing hormone (LH) [3,4]. These gonadotropins act on the ovaries, promoting follicular maturation, estradiol production, and ultimately the onset of the first ovulatory menstrual cycles. Early adolescent cycles are often anovulatory and hormonally unstable, reflecting the gradual maturation of feedback loops within the HPG axis [3]. As ovulatory function stabilizes, estrogen and progesterone levels begin to follow more predictable patterns, modulating endometrial proliferation, immune activity, and tissue remodelling within the reproductive tract [4].

From an immunological perspective, adolescence is characterized by a transitional phase in which innate and adaptive immunity recalibrate under the influence of rising sex steroids. Estrogen enhances inflammatory cytokine production, macrophage recruitment, and angiogenic signalling, while progesterone shifts immune responses toward tolerance and reduced cytotoxicity. Fluctuating estrogen-progesterone ratios typical of early adolescence may therefore amplify inflammatory responses during menstruation, creating a microenvironment that favours survival and implantation of refluxed endometrial cells in genetically susceptible girls [28].

In the peri-menarchal period, cycles are frequently anovulatory, resulting in prolonged exposure to unopposed estrogen [3,4]. This hormonal environment may further promote early lesion establishment and progression in adolescents, particularly when combined with underlying genetic or epigenetic susceptibility.

Epigenetically, puberty represents a period of heightened plasticity during which hormonal surges, environmental exposures, metabolic factors, and psychosocial stressors can induce long-lasting DNA methylation changes, histone modifications, and altered microRNA profiles. These modifications may influence genes involved in estrogen signalling, progesterone responsiveness, inflammation, extracellular matrix remodelling, and cell adhesion pathways that are central to the establishment of endometriotic lesions [29].

In parallel, genetic predisposition and inflammatory cytokine genes may lower the threshold for disease expression [9].

Together, the concurrent maturation of the HPG axis, immune modulation, pubertal epigenetic reprogramming, and inherited susceptibility may interact to create a permissive biological environment for the early initiation of endometriosis [28,29].

### 3.3. Twin Studies

Twin studies provide some of the most compelling quantitative evidence supporting the heritability of endometriosis [24,30]. A landmark analysis from the Swedish Twin Registry estimated that approximately 50% of the variance in endometriosis susceptibility can be attributed to genetic factors, underscoring the substantial contribution of inherited determinants to disease risk [55]. Consistently higher concordance rates in monozygotic compared with dizygotic twins, persisting even after adjustment for shared environmental exposures, further reinforce this genetic contribution. Notably, among discordant monozygotic twin pairs, the affected twin frequently reports earlier symptom onset, suggesting that early-onset presentations may be particularly influenced by genetic load and may represent a more penetrant expression of underlying susceptibility alleles [56].

Although large-scale twin studies specifically addressing adenomyosis are not yet available, preliminary observations point toward a possible hereditary component, especially in cases where adenomyosis coexists with endometriosis [52,53,54]. As diagnostic accuracy for both conditions improves through refined ultrasound and MRI criteria in younger individuals, studying monozygotic adolescent twins may offer a unique opportunity to elucidate early-life genetic, epigenetic and developmental contributors to disease onset [57]. This represents an important future direction for clarifying the shared and distinct heritable mechanisms underpinning adolescent-onset adenomyosis and endometriosis.

### 3.4. Genome-Wide Association Studies (GWAS)

Genome-wide association studies have substantially advanced the understanding of the polygenic nature of endometriosis, identifying a series of robust susceptibility loci across multiple populations [24,30]. Several large-scale meta-analyses have consistently highlighted variants linked to hormonal regulation, immune–inflammatory responses and cell adhesion, biological domains intimately involved in endometrial physiology and ectopic tissue survival. Among the most replicated and biologically plausible loci are WNT4 (1p36.12), a key regulator of female reproductive tract development and steroid hormone signalling [5]; ESR1 (6q25.1), encoding the estrogen receptor alpha; VEZT (12q22), associated with epithelial adhesion and tissue integrity [6]; and FSHB (11p14.1), which modulates follicle-stimulating hormone production [7,8]. These loci converge on pathways critical for estrogen sensitivity, inflammatory activation and epithelial–mesenchymal interactions, supporting the concept that variants within these genes may predispose individuals to disease beginning in early life stages [29].

However, the current GWAS landscape is heavily skewed toward adult populations, predominantly women of European ancestry, and therefore provides limited insight into the genetic determinants of adolescent-onset disease [30]. It remains unclear whether these established risk loci exert greater penetrance during puberty or whether additional, age-specific variants may interact with the hormonal and immunological transitions characteristic of adolescence [29,30]. This gap underscores the need for GWAS designed specifically for early-onset endometriosis, ideally incorporating multi-ethnic cohorts to enhance generalisability.

To date, no GWAS has been conducted exclusively on adenomyosis. Nevertheless, emerging evidence suggests partial overlap between loci implicated in endometriosis and those linked to adenomyosis or uterine fibroids, raising the possibility of shared pathogenic mechanisms [24,30]. Future integrative approaches, including combined GWAS, transcriptomic and epigenomic analyses, may help illuminate the heritable architecture of adolescent-onset adenomyosis and clarify its relationship to early-onset endometriosis.

### 3.5. Candidate Gene Studies

Beyond GWAS, candidate gene approaches have provided valuable insights into specific molecular pathways implicated in gynaecologic disorders [30,58]. In endometriosis, numerous studies have evaluated polymorphisms in genes involved in estrogen biosynthesis and metabolism, such as *CYP17A1* [59] and *COMT* [60], as well as variants in the *PGR* gene [61], inflammatory cytokines (e.g., interleukin 6 (IL-6); tumour necrosis factor (TNF-α)), and extracellular matrix remodelling enzymes, including members of the matrix metalloproteinase family (*MMPs*) [62]. Among the most frequently investigated variants, the *ESR1* (*PvuII*) and (*XbaI*) polymorphisms have been repeatedly associated with earlier onset, increased estrogen sensitivity and more severe phenotypes in several populations [6].

Nevertheless, the reproducibility of these associations remains inconsistent across studies, often due to small sample sizes, ethnic heterogeneity and methodological variability [24,30]. Additionally, very few candidate gene investigations have stratified participants by age at onset, limiting our ability to determine whether specific variants exert stronger effects during adolescence. This represents a notable gap, given that puberty is characterized by major shifts in endocrine and immune function that may unmask underlying genetic susceptibility [30].

For adenomyosis, candidate gene research is still in its early stages. Preliminary studies have suggested possible roles for *TGFB1* [63,64], *MMP2* [62] and *HOXA10* [65], particularly in pathways related to fibrosis, inflammation and endometrial–myometrial interface disruption, although the current evidence remains limited and requires validation in larger cohorts [62,63].

Overall, candidate gene findings reinforce the importance of hormone-related, inflammatory and tissue-remodelling pathways, biological processes that are highly active during puberty and therefore particularly relevant to the emergence of adolescent-onset endometriosis and adenomyosis [64,65].

## 4. Molecular Pathways and Mechanisms

### 4.1. Estrogen Signalling and Receptor Polymorphisms

Estrogen signalling plays a crucial role in the pathophysiology of both endometriosis and adenomyosis, particularly in early-onset forms where puberty-associated hormonal surges may act as a trigger in genetically predisposed individuals [6]. The biological actions of estrogen are primarily mediated through estrogen receptor α (ERα, encoded by *ESR1*) and estrogen receptor β (ERβ, encoded by *ESR2*), both abundantly expressed in endometrial and myometrial tissues [66]. Polymorphisms within these receptor genes have been associated with alterations in receptor activity, transcriptional regulation and downstream hormonal responsiveness, thereby contributing to susceptibility to estrogen-dependent disorders [67].

Among these, the *ESR1* PvuII (rs2234693) and XbaI (rs9340799) polymorphisms are among the most extensively studied in the context of endometriosis. These intronic variants may modulate mRNA stability and receptor transcriptional efficiency, ultimately influencing estrogen sensitivity at the tissue level [68,69]. Several studies, particularly in Asian populations, have reported associations between these polymorphisms and increased disease risk or greater symptom severity, although age-specific analyses in adolescent cohorts are still lacking [70,71].

Other estrogen-related genes have also been implicated. Polymorphisms in *CYP17A1* [72], a key enzyme in estrogen biosynthesis, and *COMT* [73], which regulates the metabolism of catechol estrogens, have been linked to altered local estrogen homeostasis and increased susceptibility to endometriosis [72,73]. Dysregulated estrogen metabolism within the endometrium and myometrium may amplify local estrogenic effects, promoting lesion establishment, proliferation and persistence. These mechanisms may be particularly relevant during adolescence, a developmental window characterized by heightened estrogen exposure and dynamic endocrine maturation [70,71,72].

### 4.2. Wnt/β-Catenin, PI3K/AKT/mTOR, and TGF-β Signalling Pathways

Several intracellular signalling cascades involved in embryogenesis, cellular proliferation and tissue remodelling have been implicated in the pathogenesis of endometriosis and adenomyosis [4,5]. Among these, the Wnt/β-catenin pathway is particularly noteworthy [74]. WNT4, one of the most consistently replicated susceptibility genes in GWAS for endometriosis, plays an essential role in the development of the female reproductive tract and in the regulation of mesenchymal–epithelial transitions within endometrial tissue [5,74]. Dysregulated Wnt signalling has been associated with enhanced cellular invasiveness, aberrant implantation of ectopic endometrial cells and impaired endometrial differentiation, features that may be especially relevant in early lesions emerging during adolescence [74].

The PI3K/AKT/mTOR axis represents another critical signalling pathway involved in cell growth, metabolism, angiogenesis and survival [75]. Constitutive activation of this pathway has been reported in both endometriotic and adenomyotic tissues, promoting progesterone resistance, stromal proliferation and altered apoptotic responses [76]. Genetic variants affecting the PI3K catalytic subunits or downstream AKT activation may further contribute to lesion persistence, particularly in adolescents in whom endocrine regulation is still undergoing maturation and may amplify the effects of these molecular alterations [75,76].

TGF-β signalling also exerts a multifaceted influence on tissue fibrosis, inflammation and immune modulation. Overexpression of *TGF-β1* has been consistently observed in endometriotic lesions, where it promotes fibroblast activation, excessive extracellular matrix deposition and the transition to fibrotic, deep-infiltrating phenotypes [76]. SNPs within TGFB1 have been proposed as potential risk variants, although current evidence remains preliminary and requires validation in larger cohorts [63,64]. Dysregulated TGF-β signalling may be particularly relevant in adolescents, as it intersects with pathways governing uterine development and inflammatory resolution [64].

Collectively, these signalling cascades are tightly regulated during critical windows of uterine maturation, including fetal development, neonatal life and puberty. Genetic or epigenetic dysregulation of Wnt/β-catenin [77], PI3K/AKT/mTOR [75] or TGF-β [64] pathways may therefore predispose susceptible individuals to aberrant tissue behaviour at an early age, supporting a developmental framework for adolescent-onset endometriosis and adenomyosis.

### 4.3. Inflammatory Genes and Immune Response

The inflammatory microenvironment is a defining feature of both endometriosis and adenomyosis. Numerous studies have documented aberrant expression of pro-inflammatory cytokines, including interleukin-6 (IL-6), tumour necrosis factor-alpha (TNF-α) and interleukin-1β (IL-1β), within eutopic and ectopic endometrial tissues, contributing to enhanced leukocyte recruitment, angiogenesis and nociceptive signalling [78,79,80]. Genetic polymorphisms located in the promoter regions of these cytokine genes have been associated with increased transcriptional activity, thereby amplifying the inflammatory cascade and creating a permissive environment for lesion establishment and survival [78,79].

Among the best-characterized variants, the TNF-α-308G > A (rs1800629) polymorphism has been linked to elevated circulating TNF-α levels and increased endometriosis risk, with some studies suggesting a stronger association in individuals with earlier symptom onset [80]. Likewise, the IL-6 −174G > C (rs1800795) variant has been associated with higher IL-6 expression and with more severe clinical phenotypes, including greater pain burden and more extensive disease [81].

**Figure 1:**
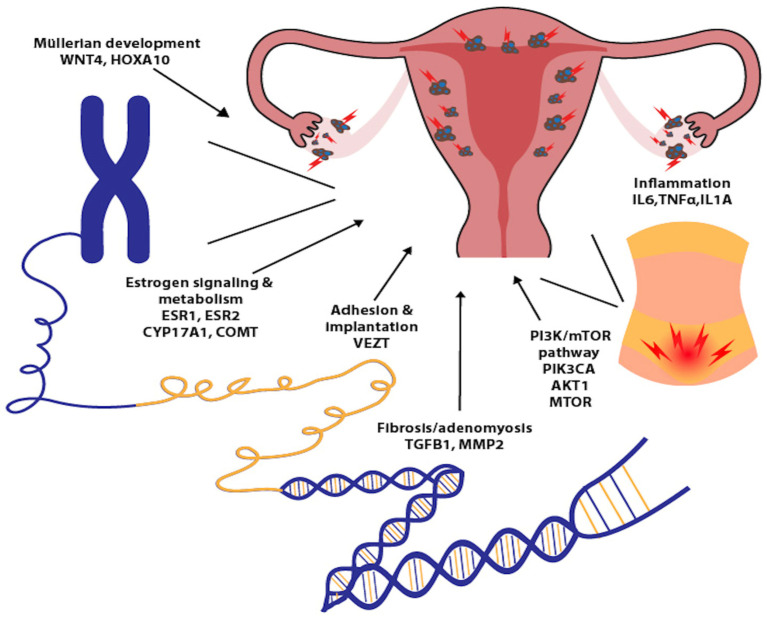
DNA architecture and its functional role in the pathogenesis of adenomyosis and endometriosis: this schematic illustration highlights how genetic variants and epigenetic modifications influence chromatin structure, transcriptional activity, and downstream inflammatory pathways.

These pro-inflammatory genotypes may exert an even greater impact during adolescence, a period marked by ongoing immune maturation and dynamic crosstalk between endocrine and immune pathways [82]. The early reproductive years are characterized by reduced efficiency of immune surveillance and altered inflammatory resolution, which may facilitate the implantation, survival and proliferation of ectopic endometrial cells in genetically predisposed individuals [81,82]. Hormonal fluctuations typical of puberty, particularly increasing estrogen levels, can further potentiate cytokine-driven inflammation, reinforcing a feedback loop that favours early lesion development (Figure 1).

The key genetic sequences and molecular mechanisms discussed in Section 3 and Section 4 are summarised in Table 1 and Table 2.

## 5. Genetic–Epigenetic–Environmental Interactions

The pathogenesis of endometriosis and adenomyosis is increasingly recognized as the result of complex interactions among genetic predisposition, epigenetic modulation and environmental exposures [85,86]. These interactions may be particularly relevant during adolescence, a period characterized by rapid endocrine maturation, immune recalibration and dynamic uterine remodelling. In individuals carrying susceptibility variants, environmental triggers acting during critical developmental windows may modulate gene expression through epigenetic mechanisms, thereby promoting early lesion initiation and persistence [86,87].

### 5.1. Epigenetic Modifications

Epigenetic mechanisms, including DNA methylation, histone modification and non-coding RNA regulation, play a pivotal role in shaping gene expression without altering the underlying DNA sequence [88,89]. In endometriosis, aberrant DNA methylation patterns have been documented in genes involved in estrogen signalling (*ESR2*) [6], progesterone responsiveness (*PGR*) [61], inflammatory regulation (*HOXA10*, *SF-1*) and cellular differentiation [17,23,65]. Such alterations may lead to dysregulated hormone sensitivity, impaired decidualization and persistent inflammatory activation, thereby facilitating ectopic endometrial survival [90,91].

Emerging studies suggest that adenomyosis also exhibits epigenetic dysregulation, particularly in genes associated with myometrial contractility, extracellular matrix remodelling, angiogenesis and tissue fibrosis [92,93,94]. Although most available epigenetic data derive from adult cohorts, early-life epigenetic programming, occurring during fetal development, neonatal life or puberty, may establish a molecular environment conducive to adolescent-onset disease [95,96].

Environmental exposures, especially endocrine-disrupting chemicals (EDCs) such as bisphenol A (BPA), dioxins and phthalates, have been shown to induce stable epigenetic changes within reproductive tissues [97,98]. These modifications may persist into adolescence and adulthood, altering susceptibility to both endometriosis and adenomyosis and providing a mechanistic link between environmental triggers and genetic risk [98].

### 5.2. Environmental Triggers and Lifestyle Factors

Multiple environmental and lifestyle factors have been implicated in modulating susceptibility to endometriosis and adenomyosis, particularly in genetically predisposed individuals. These include early menarche, low birthweight, exposure to environmental toxins, dietary patterns, and psychosocial stress [83,99,100]. For example, exposure to dioxins and polychlorinated biphenyls (PCBs) has been associated with an increased risk of endometriosis through effects on immune regulation and estrogen metabolism [101,102]. Dietary patterns rich in saturated fats and low in omega-3 fatty acids may promote a systemic pro-inflammatory milieu, whereas deficiencies in antioxidants may exacerbate oxidative stress and favour lesion survival [103,104].

In recent years, the gut microbiome has emerged as a key environmental regulator of endocrine and inflammatory homeostasis, offering a mechanistic link between lifestyle factors and disease risk. Dysbiosis may alter the enterohepatic circulation of estrogens through the activity of the *estrobolome*, the microbial β-glucuronidase system responsible for deconjugating estrogens and increasing their bioavailability, thereby amplifying the effects of genetic variants affecting estrogen receptors or metabolizing enzymes [105,106]. Evidence from recent animal and human studies indicates that endometriosis-associated dysbiosis correlates with alterations in immune–metabolic pathways, affecting T-cell and NK/NKT-cell populations, short-chain fatty acid production, and estrogen metabolite levels (106). Similarly, women with endometriosis exhibit distinct fecal microbial signatures, including enrichment of *Erysipelotrichia* and increased urinary estrogen metabolites, supporting a link between microbial enzymatic activity and estrogen recirculation [107].

From an epigenetic perspective, adolescence represents a critical window of heightened plasticity during which hormonal surges, environmental exposures, and microbiota-derived metabolites can induce durable changes in DNA methylation, histone structure, and microRNA expression [103]. These modifications may affect genes involved in estrogen signalling, progesterone responsiveness, inflammation, extracellular matrix remodelling, and cell adhesion-pathways highly relevant to adolescent-onset endometriosis and adenomyosis. Environmental disruptors such as BPA, dioxins, and phthalates are known to induce stable epigenetic alterations in reproductive tissues, further modulating disease susceptibility in genetically predisposed individuals [104,105].

Finally, psychosocial stress during puberty may alter hypothalamic–pituitary–adrenal (HPA) axis dynamics, reducing immune surveillance and enhancing inflammatory responsiveness. In adolescents with underlying genetic or epigenetic vulnerabilities, these stressors may precipitate earlier symptom onset or more aggressive disease trajectories [106,108]. Altogether, the interplay between environmental exposures, diet, microbiome–hormone interactions, and epigenetic programming provides a compelling framework to explain how adolescent biological transitions may converge with inherited susceptibility to drive early expression of endometriosis and adenomyosis [107,109].

### 5.3. Timing of Exposure and Developmental Windows

The impact of genetic, epigenetic, and environmental factors is strongly dependent on the developmental timing of exposure [110]. Adolescence represents a unique window of susceptibility, marked by profound endocrine transitions, cyclical fluctuations in estrogen and progesterone, maturation of the hypothalamic–pituitary–gonadal axis, immune recalibration, and active uterine and myometrial remodelling [84,111]. During this period, the endometrium and myometrium undergo dynamic proliferation and differentiation, while immune surveillance mechanisms gradually adapt to cyclical hormonal cues. Environmental insults or epigenetic disruptions occurring against this backdrop may therefore exert disproportionately large effects, particularly when interacting with inherited susceptibility variants that influence hormone responsiveness, inflammatory reactivity, or extracellular matrix dynamics [112,113].

Neonatal uterine bleeding (NUB) has been proposed as an early-life mechanistic trigger capable of generating a developmental origin for later disease. In genetically predisposed individuals, retrograde flow of neonatal endometrial cells into the peritoneal cavity or their entrapment within the myometrium may establish ectopic cell populations that remain quiescent until activated by pubertal hormonal surges, thereby facilitating the emergence of adenomyosis or endometriosis during adolescence [112].

Similarly, prenatal or early postnatal exposure to endocrine-disrupting chemicals such as bisphenol A, dioxins, and phthalates may prime the epigenetic landscape by altering DNA methylation, histone modification, or microRNA expression in reproductive tissues [97]. In animal models, in utero exposure to TCDD has been shown to induce adenomyosis-like phenotypes [101], whereas human data, although more limited, suggest that most documented exposures occur prenatally rather than postnatally [113]. These early-life epigenetic signatures may persist into puberty, lowering the threshold for lesion establishment once cyclical estrogen exposure intensifies [113].

Beyond endocrine factors, adolescence is characterized by shifts in immune tolerance, macrophage polarization, T-cell maturation, and inflammatory resolution pathways, all of which influence tissue homeostasis and may facilitate ectopic implantation or persistence of refluxed endometrial cells. The convergence of pubertal hormonal surges with an immune system still in transition may enhance inflammatory amplification loops, particularly in individuals carrying variants in cytokine-related genes, estrogen receptors, or matrix remodelling pathways. These processes may further be shaped by external triggers such as diet, stress, microbiome composition, and early life metabolic profiles [114].

Taken together, the interplay between inherited genetic risk, developmental physiology, epigenetic priming, and environmental exposures may determine not only whether disease occurs, but also its age of onset, anatomical distribution, phenotypic severity, and response to therapy. This developmental framework underscores the urgent need for adolescent-focused studies exploring the timing, magnitude, and mechanistic pathways of gene–environment interactions, particularly those acting during critical windows such as neonatal life, prepubertal years, and early puberty [115,116] (Table 3).

## 6. Clinical Implications and Future Directions

The recognition of a strong genetic and molecular basis for adolescent-onset endometriosis and adenomyosis carries important clinical implications. Reframing these conditions not merely as acquired disorders but as developmentally influenced diseases with substantial hereditary components opens new perspectives for early diagnosis, risk stratification and personalized management [115,116,117].

### 6.1. Toward Early Identification of High-Risk Individuals

Identifying genetic variants associated with early-onset disease may eventually enable preclinical risk assessment in adolescent girls with a positive family history or early suggestive symptoms [43]. For example, daughters or sisters of women with confirmed endometriosis or adenomyosis could be prioritized for closer clinical monitoring during puberty, especially if presenting with severe or refractory dysmenorrhea, an age group in which diagnostic delays remain common [116].

Although routine genetic screening is not yet recommended, advances in polygenic risk scoring and the integration of genetic markers with clinical data may support the development of predictive algorithms. Such tools could help stratify adolescents at higher risk of disease progression and guide early imaging, timely initiation of medical therapy or referral to specialized care [118,119].

### 6.2. Personalizing Treatment and Prognosis

Understanding the molecular and genetic underpinnings of these conditions may inform personalized therapeutic decisions. For instance, variants within estrogen or progesterone receptor genes have been linked to differential hormonal responsiveness. Adolescents with polymorphisms associated with progesterone resistance may benefit from earlier adoption of non-hormonal or second-line treatments [47].

Similarly, aberrant activation of signalling pathways such as Wnt/β-catenin or PI3K/mTOR could, in the future, guide the use of targeted pharmacological interventions in individuals with refractory symptoms. While these approaches remain experimental, ongoing progress in molecular profiling suggests that pathway-directed therapy may eventually become integrated into clinical practice [117].

From a prognostic standpoint, adolescents with a higher genetic burden may be predisposed to more extensive lesions, multi-compartment involvement or higher recurrence rates. Recognizing these patterns early may help clinicians tailor follow-up strategies, counsel families more effectively and initiate timely fertility-preservation planning when appropriate [118].

### 6.3. Gaps in Knowledge and Research Priorities

Despite growing evidence, substantial knowledge gaps persist. Most genetic and epigenetic studies have focused on adult women, with minimal adolescent-specific data available [13,43]. There is a clear need for longitudinal cohort studies beginning in early adolescence that track genetic, environmental and clinical variables over time. Such studies could clarify whether early-onset disease represents a distinct molecular entity or an earlier expression of the same pathogenic continuum observed in adults [32,33,47].

Key research priorities include

- Adolescent-focused GWAS and epigenome-wide association studies (EWAS) [30];
- Integration of multi-omics datasets (genomics, transcriptomics, proteomics, metabolomics) [120];
- Detailed investigation of gene–environment interactions across critical developmental windows [78];
- Inclusion of diverse ethnic backgrounds to improve generalisability and equity in precision medicine [86].

Ultimately, bridging the gap between molecular science and clinical care will require sustained interdisciplinary collaboration among gynecologists, geneticists, epidemiologists, pediatric specialists and bioinformaticians [121].

### 6.4. Strengths and Limitations

This review provides a comprehensive synthesis of the genetic, epigenetic and environmental contributors to adolescent-onset endometriosis and adenomyosis. A major strength lies in its integrative approach, combining evidence across molecular, developmental and clinical domains to contextualize these disorders within a unified pathogenic framework. The inclusion of recent genomic and epigenomic studies ensures an updated and evidence-based perspective.

Nevertheless, limitations must be acknowledged. The majority of available research is derived from adult cohorts, with few studies specifically focused on adolescents. Heterogeneity in study design, diagnostic criteria and sample size limits comparability across studies. Additionally, functional validation of many genetic and epigenetic findings remains incomplete, and environmental exposures, such as endocrine disruptors, diet or psychosocial stress, are inconsistently characterized. Finally, as a narrative review, the absence of a formal quality appraisal may introduce selection bias.

### 6.5. Future Perspectives

Future research should focus on elucidating the developmental origins of endometriosis and adenomyosis through adolescent-centred approaches [120]. Longitudinal cohort studies initiated early in life will be essential to clarify temporal relationships between genetic variants, epigenetic reprogramming and environmental influences. Multi-omics integration, including single-cell sequencing and spatial transcriptomics—may reveal distinct molecular endotypes that improve diagnostic precision and patient stratification [121].

Advances in polygenic risk scoring and molecular profiling could ultimately support personalized prevention strategies, early detection of high-risk adolescents and targeted therapeutic interventions. Strengthening collaboration among gynecologists, pediatricians, geneticists and computational scientists will be fundamental to translating basic research into clinically actionable insights and advancing precision gynecology [120,121].

## 7. Conclusions

Adolescent-onset endometriosis and adenomyosis are increasingly recognized as clinically significant and potentially distinct phenotypes with strong genetic and molecular determinants. Evidence from familial studies, GWAS and candidate gene analyses suggests that hereditary factors, interacting with hormonal, immune and environmental influences during critical developmental windows, play a major role in early disease initiation.

Although most current data are derived from adult populations, growing interest in adolescent-specific research underscores the urgent need for studies designed for this age group. A deeper understanding of the genetic and epigenetic landscape of early-onset disease may support earlier diagnosis, refined risk prediction and more personalized management strategies for affected adolescents.

Future research integrating genomic, epigenomic and environmental data will be essential to unravel the complex etiology of these conditions and to translate molecular insights into meaningful clinical benefits.

## Figures and Tables

**Table 1:** Main genetic loci associated with endometriosis and their biological functions.

Gene/Locus	Chromosomal Location	Biological Function	Pathway Involved	Evidence Type
WNT4 [5,74]	1p36.12	Female reproductive tract development; steroid hormone signalling	Wnt/β-catenin pathway	GWAS, functional studies
ESR1 [66,67,68]	6q25.1	Estrogen receptor alpha; modulates estrogen response in endometrial tissue	Estrogen signalling	GWAS, candidate gene studies
FSHB [8]	11p14.1	Follicle-stimulating hormone regulation	HPO axis regulation	GWAS
VEZT [7]	12q22	Cell adhesion and epithelial stability	Cell junctions	GWAS
GREB1 [6]	2p25.1	Estrogen-responsive gene; promotes endometrial cell proliferation	Estrogen-mediated transcription	GWAS
IL1A [4]	2q14.1	Pro-inflammatory cytokine; promotes local inflammation	Inflammatory response	GWAS

GWAS, genome-wide association study; HPO, hypothalamic–pituitary–ovarian; FSHB, follicle-stimulating hormone beta; ESR1, estrogen receptor 1; IL1A, interleukin 1 alpha; VEZT, vezatin.

**Table 2:** Candidate gene polymorphisms associated with early-onset endometriosis or adenomyosis.

Gene	Variant/SNP	Associated Condition	Functional Effect	Population Studied	Type of Evidence	Key References
ESR1	rs2234693 (PvuII)	Endometriosis	Modulates ESR1 transcription; impacts estrogen responsiveness	Asian, European	Multiple human case–control studies + meta-analyses	[6,66,67,68]
ESR1	rs9340799 (XbaI)	Endometriosis	Alters ESR1 expression and ER-dependent signalling	Asian, European	Human candidate gene studies	[69,70,83]
CYP17A1	rs743572	Endometriosis	Increases estrogen biosynthesis	Mixed (mainly Asian & Caucasian cohorts)	Human functional genetics + case–control	[59,71,73]
COMT	rs4680 (Val158Met)	Endometriosis	Decreases COMT activity: increased catechol-estrogens	Asian, European	Human case–control and meta-analyses	[60]
PGR	+331G/A	Endometriosis	Reduces PR-B expression: progesterone resistance	Mixed	Human case–control	[61]
HOXA10	Hypermethylation	Endometriosis	Silencing of HOXA10: impaired implantation & uterine differentiation	Mixed	Human epigenetic studies	[65,84]
ERβ pathway	NA	Endometriosis	Aberrant overexpression of ERβ modulating inflammation & apoptosis	Animal + human tissue studies	Mechanistic in vivo (ER-β KO mouse) + ex vivo	[67]
ESR1/ESR2 (general function)	NA	NA	Physiological roles of ERα/ERβ defined in knockout mice	Animal model	Mouse knockout studies	[66]

SNP, single-nucleotide polymorphism; ESR1, estrogen receptor alpha; CYP17A1, cytochrome P450 family 17 subfamily A member 1; COMT, catechol-O-methyltransferase; PGR, progesterone receptor; HOXA10, homeobox A10; NA: Not Applicable.

**Table 3:** Gene–environment interactions and developmental windows implicated in adolescent endometriosis and adenomyosis.

Genetic Factor	Environmental Trigger	Proposed Mechanism	Developmental Window	Potential Outcome
ESR1 polymorphisms [6,66,67,68,69,83]	High-fat diet, endocrine disruptors	Enhanced estrogen sensitivity, disrupted receptor function	Puberty (hormonal surge)	Early symptom onset, severe dysmenorrhea
WNT4 variants [5,74]	Neonatal uterine bleeding (NUB)	Aberrant Müllerian duct remodelling	Neonatal period	Myometrial invasion, adenomyotic foci
IL-6/TNF polymorphisms [62]	Infections, inflammatory diet	Heightened inflammatory response	Childhood–adolescence	Chronic pelvic pain, immune dysregulation
HOXA10 methylation [65,84]	BPA exposure, dioxins	Epigenetic silencing of implantation-related genes	In utero–puberty	Impaired endometrial receptivity, ectopic implantation
COMT variants [60,73]	Oxidative stress, low antioxidant intake	Accumulation of estrogen metabolites	Adolescence	Local estrogen excess, lesion persistence

BPA, bisphenol A; ESR1, estrogen receptor 1; HOXA10, homeobox A10; COMT, catechol-O-methyltransferase; IL-6, interleukin 6; TNF, tumour necrosis factor; NUB, neonatal uterine bleeding.

## Data Availability

The present review was based on published articles. All summary data generated during this study are included in this published article. Raw data used for the analyses are available presented in the original reviewed articles.

## Abbreviations

BPA	Bisphenol A
COMT	Catechol-O-methyltransferase
DIE	Deep Infiltrating Endometriosis
EDC	Endocrine-Disrupting Chemical
ERα/ERβ	Estrogen Receptor alpha/beta
ESR1/ESR2	Estrogen Receptor 1/2 (genes encoding ERα/ERβ)
EWAS	Epigenome-Wide Association Study
FSHB	Follicle-Stimulating Hormone Beta
GWAS	Genome-Wide Association Study
HPA axis	Hypothalamic–Pituitary–Adrenal axis
HPO axis	Hypothalamic–Pituitary–Ovarian axis
HOXA10	Homeobox A10
IL-1β/IL-6	Interleukin-1 beta/Interleukin-6
IL1A	Interleukin 1 alpha
mTOR	Mechanistic Target of Rapamycin
NUB	Neonatal Uterine Bleeding
PCBs	Polychlorinated Biphenyls
PGR	Progesterone Receptor
PI3K	Phosphoinositide 3-Kinase
SNP	Single-Nucleotide Polymorphism
TGF-β	Transforming Growth Factor Beta
TNF-α	Tumour Necrosis Factor Alpha
VEZT	Vezatin
WNT4	Wingless-Type MMTV Integration Site Family Member 4
